# External validation study of endometrial cancer preoperative risk stratification model (ENDORISK)

**DOI:** 10.3389/fonc.2022.939226

**Published:** 2022-08-03

**Authors:** Petra Vinklerová, Petra Ovesná, Jitka Hausnerová, Johanna M. A. Pijnenborg, Peter J. F. Lucas, Casper Reijnen, Stephanie Vrede, Vít Weinberger

**Affiliations:** ^1^ Department of Gynecology and Obstetrics, University Hospital Brno and Faculty of Medicine, Masaryk University, Brno, Czechia; ^2^ Institute of Biostatistics and Analyses, Faculty of Medicine, Masaryk University, Brno, Czechia; ^3^ Department of Pathology, University Hospital Brno and Faculty of Medicine, Masaryk University, Brno, Czechia; ^4^ Department of Obstetrics and Gynecology, Radboud University Medical Center, Nijmegen, Netherlands; ^5^ Department of Data Science, University of Twente, Enschede, Netherlands; ^6^ Department of Radiation Oncology, Radboud University Medical Center, Nijmegen, Netherlands

**Keywords:** Bayesian networks model, disease-specific survival, endometrial cancer, prognosis, risk stratification, sentinel node biopsy, lymph node metastasis

## Abstract

**Introduction:**

Among industrialized countries, endometrial cancer is a common malignancy with generally an excellent outcome. To personalize medicine, we ideally compile as much information as possible concerning patient prognosis prior to effecting an appropriate treatment decision. Endometrial cancer preoperative risk stratification (ENDORISK) is a machine learning–based computational Bayesian networks model that predicts lymph node metastasis and 5-year disease-specific survival potential with percentual probability. Our objective included validating ENDORISK effectiveness in our patient cohort, assessing its application in the current use of sentinel node biopsy, and verifying its accuracy in advanced stages.

**Methods:**

The ENDORISK model was evaluated with a retrospective cohort of 425 patients from the University Hospital Brno, Czech Republic. Two hundred ninety-nine patients were involved in our disease-specific survival analysis; 226 cases with known lymph node status were available for lymph node metastasis analysis. Patients were included undergoing either pelvic lymph node dissection (*N* = 84) or sentinel node biopsy (*N* =70) to explore the accuracy of both staging procedures.

**Results:**

The area under the curve was 0.84 (95% confidence interval [CI], 0.77–0.9) for lymph node metastasis analysis and 0.86 (95% CI, 0.79–0.93) for 5-year disease-specific survival evaluation, indicating quite positive concordance between prediction and reality. Calibration plots to visualize results demonstrated an outstanding predictive value for low-risk cancers (grades 1–2), whereas outcomes were underestimated among high-risk patients (grade 3), especially in disease-specific survival. This phenomenon was even more obvious when patients were subclassified according to FIGO clinical stages.

**Conclusions:**

Our data confirmed ENDORISK model’s laudable predictive ability, particularly among patients with a low risk of lymph node metastasis and expected favorable survival. For high-risk and/or advanced stages, the ENDORISK network needs to be additionally trained/improved.

## Introduction

In industrialized countries, endometrial cancer (EC) is a common malignancy with generally an excellent outcome and 5-year relative survival rate of 76% among European women (1). Despite its overall favorable prognosis, up to 15% of patients classified as low-risk will experience recurrence and may profit from adjuvant treatment (2). Conversely, a substantial number of patients classified as high-risk surprisingly evidence no disease recurrence many years after treatment. Respecting the current emphasis on personalized medicine, we ideally seek as much information as possible concerning a patient’s prognosis prior to determine the most effective therapeutic approach, avoid overtreatment, and prevent treatment-related morbidity. Current European guidelines classify patients into five prognostic risk groups based on final tumor stage and histological characteristics (3). However, in the preoperative setting, risk stratification can be challenging owing to the lack of certain essential definitive histology information such as lymphovascular space invasion (LVSI) and myometrial invasion.

Lymph node (LN) involvement is an important issue that impacts treatment approach and is related to poor prognosis. Two large randomized trials (4, 5) renounced the curative significance of lymphadenectomy. Nowadays, pelvic and para-aortic lymphadenectomy (PLN and PALN) are mainly considered as staging tools with substantial morbidity (6). According to the recent European guidelines, sentinel node biopsy (SNB) is an alternative to full lymphadenectomy in low/intermediate-risk stage I/II EC and can also be considered in high-intermediate and high-risk stage I/II groups (3).

In order to identify preoperatively which patients are at risk for lymph node metastasis (LNM), the endometrial cancer preoperative risk stratification (ENDORISK) was constructed within the ENITEC network (European Network of Individal Treatment in Endometrial Cancer) (7). This is a machine learning–based computational Bayesian networks model, which predicts the probability of LNM and 5-year disease specific survival (DSS) in EC cases. This ENDORISK model has been validated forthwith using two multicentric cohorts: MoMaTEC (the Molecular Markers in Treatment in Endometrial Cancer) (8) and PIPENDO (the PIpelle Prospective ENDOmetrial carcinoma) study (9). The diagnostic accuracy was 0.82 and 0.84, respectively. Input data contains preoperative clinical and histological characteristics. Since the original model consisted of a notably heterogeneous patient group from many countries with possible treatment decision divergencies, we were questioning how this model would perform within our patient cohort with very well-structured and collected preoperative clinical/histological data, adjuvant treatment, and follow-up.

Our aim was to validate the ENDORISK model’s accuracy and the applicability within the current SNB staging era. Since the model was constructed based on full lymphadenectomy, our further objective was to evaluate the model’s potential accuracy bias by introducing the SNB method. Additionally, we wanted to verify the model’s performance within advanced EC stages. Our study points out the weaknesses and strengths of the original ENDORISK model and proposes certain modifications in order to utilize the model within the actual and real clinical practice worldwide.

## Methods

### Patient cohort

We evaluated the ENDORISK model in our retrospectively collected study cohort including 425 patients treated at the University Hospital Brno, Czech Republic. Our cohort evolved from an EC database of 835 patients treated between January 2006 and May 2021. Cases that were incorporated in the original ENDORISK model (*N* = 150) and those without the minimally required data for using ENDORISK (*N* = 240) were excluded.

We assessed clinical and histological characteristics from the EC database and patients’ medical records: age, BMI, follow-up length, preoperative tumor grade/histotype, estrogen receptor (ER), progesterone receptor (PR), L1 Cell Adhesion Molecule (L1CAM), p53 expression, cancer antigen (Ca) 125 serum level, platelet count, preoperative cervical cytology result, lymphadenopathy according to imaging methods, myometrial/cervical invasion, LVSI, clinical/surgical staging, LN staging method, LNM, and adjuvant treatment.

All patients underwent preoperative biopsy *via* hysteroscopy or dilatation and curettage, imaging staging procedures with expert ultrasound and computed tomography (CT) scan to detect local or distant disease spread, and retroperitoneal lymphadenopathy. Patients were allocated to the clinical FIGO (International Federation of Gynecology and Obstetrics) (2009) stages. Subsequently, patients were classified into low- and high-risk groups. The low-risk group was defined as endometrioid/mucinous carcinoma, clinically FIGO stage 1A or 1B, grade 1; and endometrioid/mucinous cancer clinically FIGO 1A, grade 2, all without clinical or imaging evidence of lymphadenopathy or distant metastases. When the low-risk criteria were not met, patients were considered high risk.

### Surgical treatment

Hysterectomy with bilateral salpingo-oophorectomy as basic surgical treatment was performed with an abdominal or laparoscopic approach. In addition, high-risk patients underwent systematic para-aortic/pelvic lymphadenectomy (historically pelvic lymphadenectomy only)—at least five LNs from each hemipelvis and 10 from the para-aortic region were removed. Since 2019, systematic lymphadenectomy has been replaced by SNB in all EC patients regardless of their preoperative risk group. Currently, lymphadenectomy is limited to patients experiencing bulky LNs on preoperative imaging or perioperative finding.

### Sentinel node ultrastaging

Regarding sentinel node methodology, we used intracervical indocyanine green injections and searched for the nodes with an endoscopic fluorescence imaging camera (Novadaq Pinpoint).

All sentinel LNs were fixed in 10% buffered formalin, sliced at 2-mm lamellas, embedded in paraffin, and further examined by ultrastaging protocol. This protocol consists of two consecutive 4-μm thick sections obtained in regular 200-μm intervals, which are cut from each paraffin block. The first section was stained with hematoxylin and eosin, and the second section was examined with cytokeratins (AE1/3). We classified micrometastasis (0.2–2 mm) together with macrometastases (>2 mm) as LN positive, whereas isolated tumor cells (≤0.2 mm or single cells/clusters of cells ≤200 cells in a single LN cross-section) were considered LN negative.

### Immunohistochemical analysis

The experienced gynecological histopathologist (J. H.) examined all hematoxylin and eosin-stained slides to confirm preoperative histological subtype and grade. Immunohistochemical staining was effected on formalin-fixed and paraffin-embedded tissue sections. L1CAM positivity was defined as distinct membrane staining in ≥10% of tumor cells. ER and PR were considered positive when there were ≥10% of tumor cells with nuclear staining. p53 was classified into wild type or mutant (strong diffuse overexpression in more than 90% of tumor cells or completely negative) phenotypes.

### Statistical analysis

Following the original ENDORISK model validation, we used preoperative tumor grade, at least three IHC markers (ER, PR, p53, or L1CAM) and at least one of the clinical preoperative markers (CA 125 serum level, LN status according to imaging method, platelet count, or pap smear result) as the minimal input data. A five-year follow-up (in the 5-year DSS group) and LN staging procedure (in the LNM group) were available in all included cases.

Probabilities of LNM and 5-year DSS were calculated for each patient and compared with observed reality. (i) Discrimination testing was assessed using a receiver operating characteristic (ROC) curve generated by plotting sensitivity against 1-specificity. Discriminating performance was quantified based on the AUC (area under the curve). (ii) The model’s overall performance was quantified by the Brier score, which is the mean squared difference between each predicted probability and the observed outcome; a lower Brier score indicates better accuracy of probabilistic predictions. (iii) Calibration was visualized using a calibration plot, in which the predicted outcome was plotted against the observed outcome. To quantify model calibration, the predicted number of events (i.e., sum of each predicted probability) was compared with the observed number. (iv) Concordance between the ENDORISK model, our data, and recent DSS prediction was undertaken by using U.K. Uterine cancer survival data for different FIGO stages (10). Sensitivity analysis was accomplished by omitting patients with only SNB. Analyses were achieved in R (4.1.1) with the bnlearn (4.7), pROC (1.18.0), DescTools (0.99.44), and caret (6.0–90) packages.

### Ethics approval

Our study was approved by the University Hospital Brno Ethics Committee, Approval Number 06-151221/EK. All patients signed informed consent for histology sample storage, scientific use, and publication purposes.

## Results

**Figure 1:**
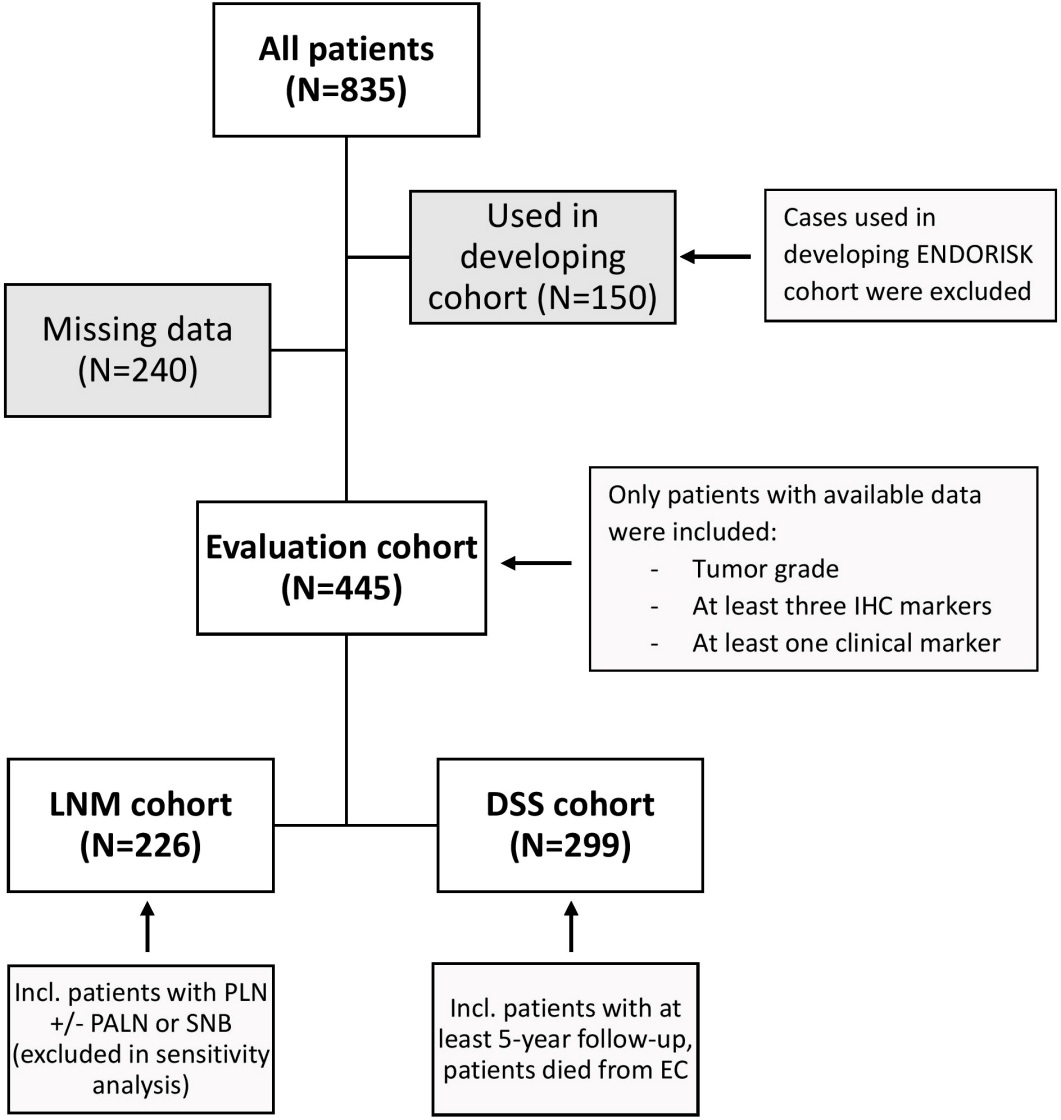
Cohort development. The evaluation cohort was developed using all patients from our clinical database treated between January 2006 and May 2021 with available data and lymph node staging (LNM cohort) and/or 5-year follow-up (DSS cohort). IHC, immunohistochemical; LNM, lymph node metastasis; DSS, disease specific survival; PLN, pelvic lymphadenectomy; PALN, para-aortal lymphadenectomy; SNB, sentinel node biopsy; EC, endometrial cancer.

Among the 835 patients in our EC database treated between January 2006 and May 2021, 299 patients were involved in our DSS analysis; 226 cases were available for LNM analysis (**Figure 1**). **Table 1** summarizes clinical data, histological characteristics, and adjuvant treatment.

**Table 1:** Clinical and histological characteristics.

Variable		LNM cohort	5- year DSS cohort
Total N		N = 226*	N = 299*
Age (years)		64.5 (59.0 to 68.8)	65.0 (59.0 to 72.0)
BMI (kg/m^2^)		30.0 (26.0 to 34.0)	32.0 (27.0 to 36.0)
Follow up length (month)		35.2 (13.0 to 90.6)	91.8 (64.5 to 122.2)
Preoperative tumor grade	1	38 (16.8)	62 (20.7)
	2	103 (45.6)	171 (57.2)
	3	85 (37.6)	66 (22.1)
ER expression	Negative	28 (12.4)	23 (7.7)
	Positive	198 (87.6)	276 (92.3)
PR expression	Negative	42 (18.6)	38 (12.7)
	Positive	184 (81.4)	261 (87.3)
L1CAM expression	Negative	175 (77.4)	258 (86.3)
	Positive	48 (21.2)	40 (13.4)
	Unknown	3 (1.3)	1 (0.3)
p53 expression	Wild type	185 (81.9)	244 (81.6)
	Muttated	37 (16.4)	37 (12.4)
	Missing	4 (1.8)	18 (6.0)
Ca-125	Negative (<35)	167 (73.9)	195 (65.2)
	Positive (35+)	47 (20.8)	63 (21.1)
	Unknown	12 (5.3)	41 (13.7)
Trombocytosis	No	215 (95.1)	279 (93.3)
	Yes	7 (3.1)	11 (3.7)
	Unknown	4 (1.8)	9 (3.0)
Imaging results	No lymphadenopathy	210 (92.9)	271 (90.6)
	Lymphadenopathy	11 (4.9)	10 (3.3)
	Unknown	5 (2.2)	18 (6.0)
Cervical cytology	Normal	143 (63.3)	200 (66.9)
	Abnormal	7 (3.1)	4 (1.3)
	Unknown	76 (33.6)	95 (31.8)
Histological subtype	Endometrioid	186 (82.3)	275 (92.0)
	Non-endometrioid	40 (17.7)	24 (8.0)
Myometrial invasion	less then 50%	129 (57.1)	200 (66.9)
	more then 50%	97 (42.9)	99 (33.1)
Cervical invasion	No	193 (85.4)	266 (89.0)
	Yes	33 (14.6)	33 (11.0)
FIGO stage (surgical)	IA	108 (47.8)	180 (60.2)
	IB	41 (18.1)	58 (19.4)
	II	29 (12.8)	30 (10.0)
	IIIA	5 (2.2)	9 (3.0)
	IIIB	1 (0.4)	1 (0.3)
	IIIC	38 (16.8)	16 (5.4)
	IV	4 (1.8)	5 (1.7)
LVSI	No	170 (75.2)	271 (90.6)
	Yes	53 (23.5)	24 (8.0)
	Unknown	3 (1.3)	4 (1.3)
Type of lymphadenectomy	PLN	84 (37.2)	68 (22.7)
	PLN+PALN	72 (31.9)	31 (10.4)
	SNB	70 (31.0)	1 (0.3)
	Unknown	0 (0.0)	199 (66.6)
Lymph nodes	Negative	185 (81.9)	87 (29.1)
	Positive	41 (18.1)	17 (5.7)
	Unknown	0 (0.0)	195 (65.2)
SNB	Negative	65 (28.8)	1 (0.3)
	Positive	5 (2.2)	0 (0.0)
	Unknown	156 (69.0)	298 (99.7)
Adjuvant treatment	None	84 (37.2)	163 (54.5)
	RT	94 (41.6)	106 (35.5)
	CHT	17 (7.5)	14 (4.7)
	CHRT	27 (11.9)	9 (3.0)
	Unknown	4 (1.8)	7 (2.3)

*n (%); Median (IQR).

DSS, disease-specific survival; LNM, lymph node metastasis; BMI, Body Mass Index; ER, Estrogen receptor; PR, Progesterone receptor; L1CAM, L1 cell adhesion molecule; LVSI, lymphovascular space invasion; PLN, pelvic lymphadenectomy; PALN, para-aortic lymphadenectomy; SNB, sentinel node biopsy; RT, radiotherapy; CHT, chemotherapy; CHRT, chemoradiotherapy.

### LNM analysis

A total of 226 patients were included in our LNM analysis: 84 (37%) PLN, 72 (32%) PLN+PALN, and 70 (31%) SNB. Forty-one patients had at least one LNM (18%): 24 (59%) in pelvic, three (7%) in para-aortic, and 14 (34%) in both localizations. A median of 27 and 22 LNs were removed during PLN and PALN, respectively.

**Figure 2:**
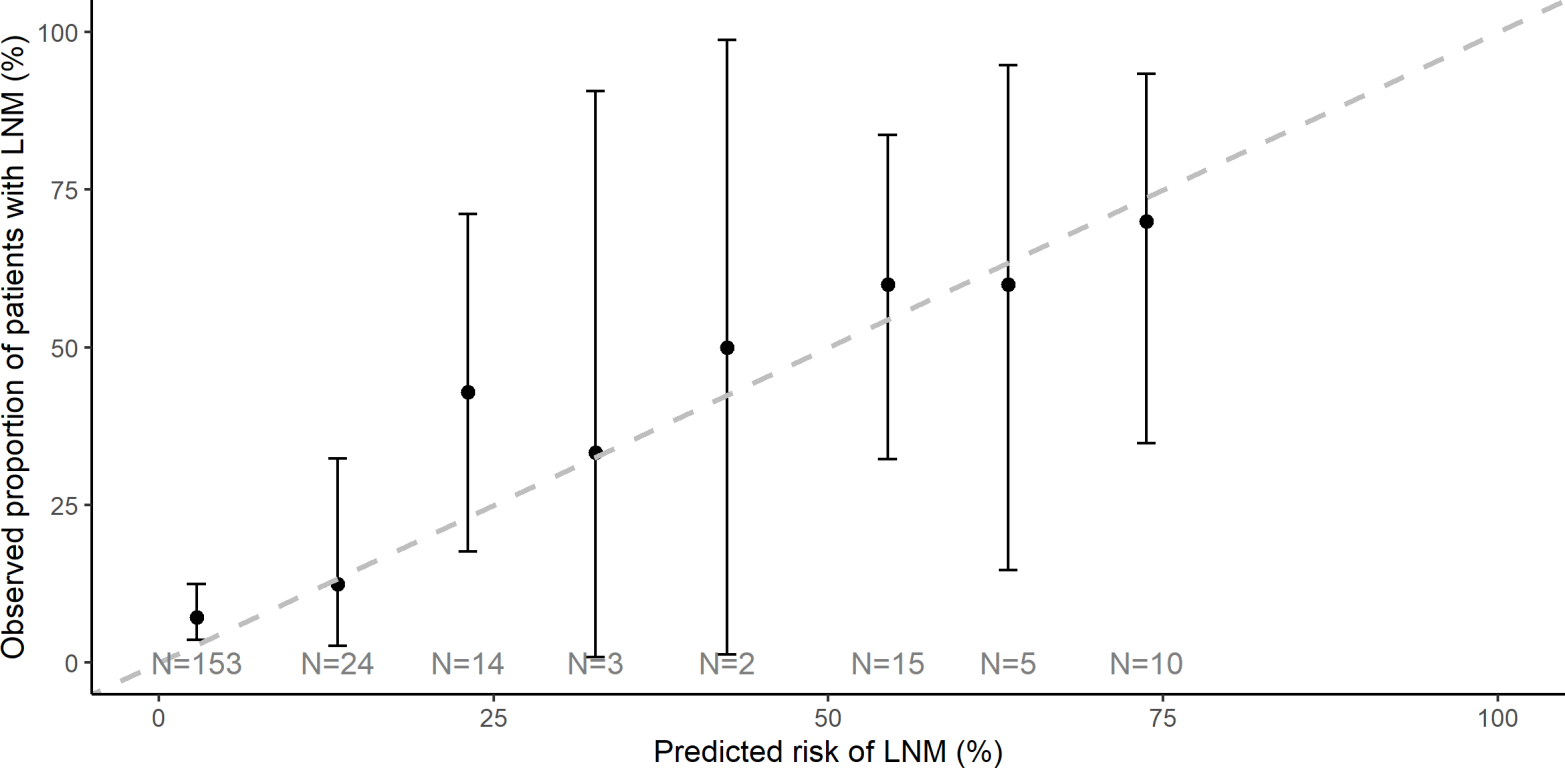
Lymph node metastasis calibration plot of observed versus predicted events. A dashed line displays the predicted value, and black dots represent the observed LNM. Ideally, all black marks are lying on the dashed line. LNM, lymph node metastasis.

The AUC (0.84) and Brier score (0.11) indicated good concordance between prediction and reality (**Table 2**, **Figure 2**). Predicted/observed ratio displayed non-significant underestimation (0.76; 95% CI 0.49–1.03). Results from sensitivity analysis, where cases with SNB were excluded, were comparable (**Supplementary Material: Table 1**, **Figure 1**), indicating that involvement in the main analysis did not alter the accuracy of ENDORISK.

**Table 2:** Model concordance statistics.

	LNM	5-year DSS
AUC (95% CI)	0.84 (0.77-0.9)	0.86 (0.79-0.93)
Brier score	0.11	0.09
Predicted no. of events	31.2	271.7
Observed no. of events	41	262
Predicted/observed ratio (95% CI)	0.76 (0.49-1.03)	1.04 (0.91-1.16)

Both AUC and Brier score substantiate very good concordance between prediction and reality in general across the dataset. Discriminative performance was quantified based on AUC (a higher AUC indicates better performance). Overall model performance was quantified by the Brier score (a lower Brier score characterizes better accuracy of the probabilistic predictions).

The predicted/observed ratio <1 denotes a lower prediction than reality, whereas a ratio >1 signals overestimation compared with reality. If 95% CI includes value 1, the difference is non-significant.

AUC, area under the curve; CI, confidence interval; DSS, disease specific survival; LNM, lymph node metastasis.

**Figure 3:**
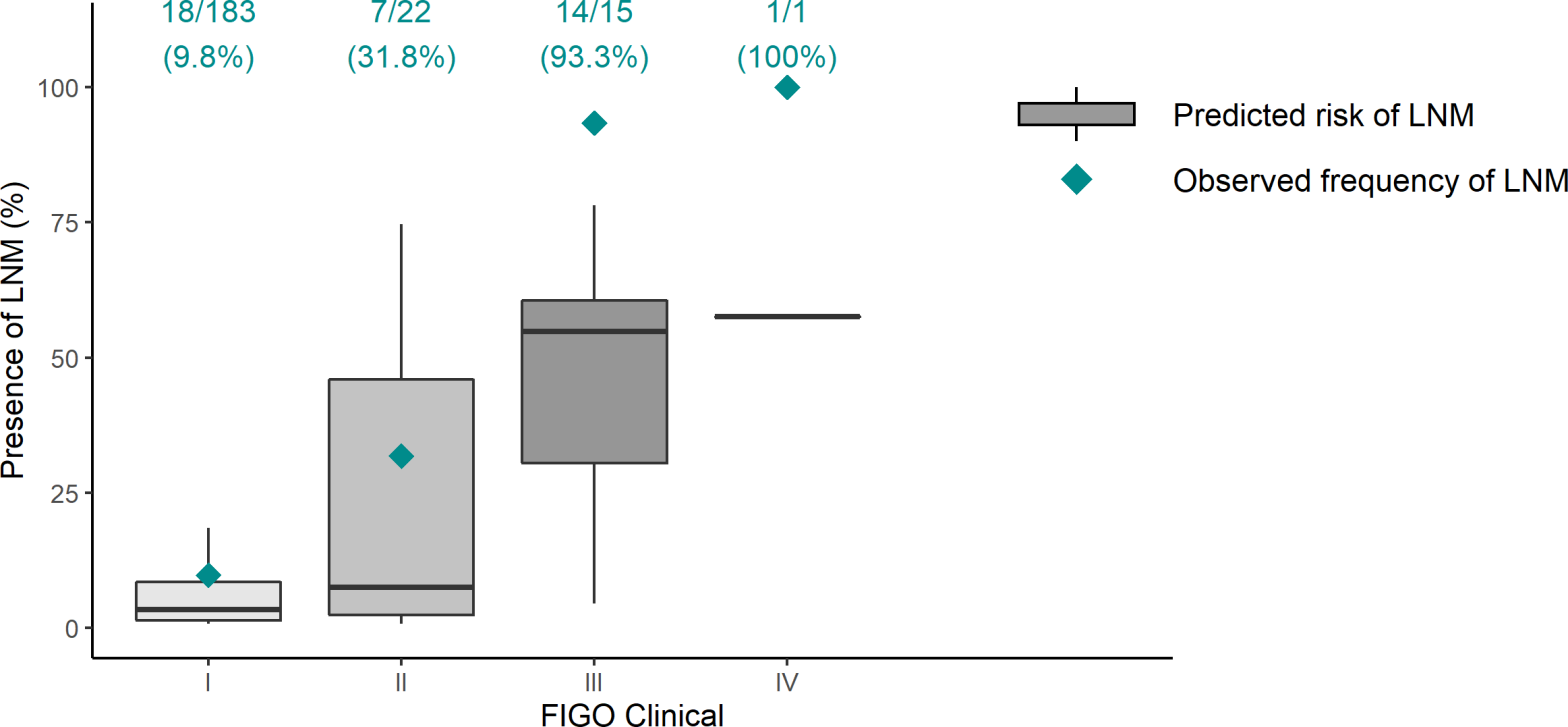
Lymph node metastasis prediction versus reality in different clinical FIGO stages. Gray boxes represent the model’s prediction; green rhombuses indicate the real LNM frequency. Ideally, all green rhombuses lie in gray boxes. In clinical FIGO III–IV stages, ENDORISK predicts fewer cases of LNM than reality. LNM, lymph node metastasis; FIGO, International Federation of Gynecology and Obstetrics.

**Figure 3** shows LNM prediction and reality for the different clinical FIGO stages (**Supplementary Figure 2** complements surgical stages).

### DSS analysis

**Figure 4:**
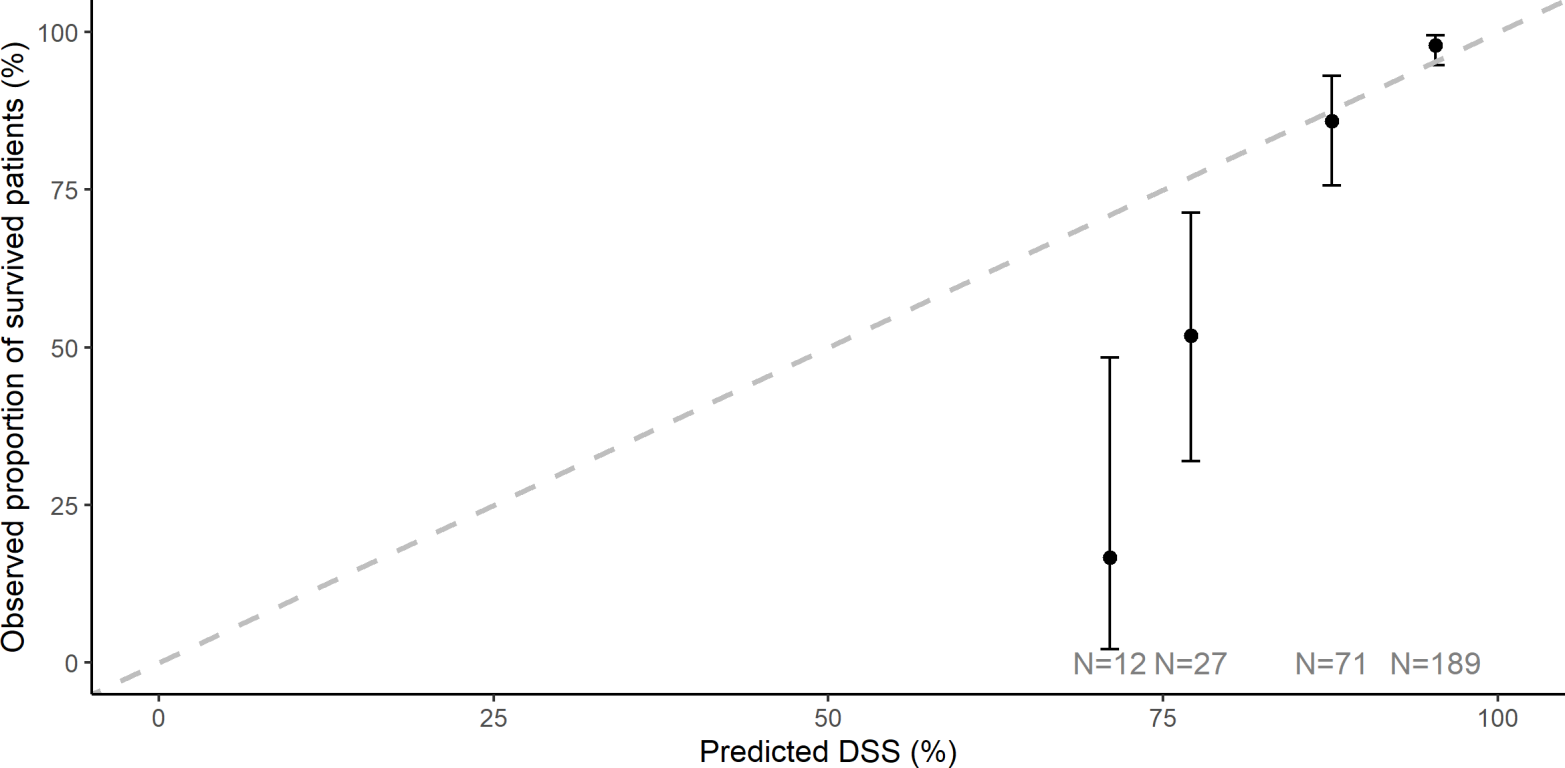
Five-year disease-specific survival calibration plot of observed versus predicted events. A dashed line displays the predicted value; black dots represent observed DSS. Ideally, all black marks are lying on the dashed line. There is a trend toward overestimating survival in the lower predicted survival rates. DSS, disease-specific survival.

Only patients with at least 5 years of follow-up or who died from EC were included (*N* = 299). The AUC was 0.86 (95% CI, 0.79–0.93), Brier score 0.09. Five-year DSS prediction was well calibrated with a trend toward overestimating survival among the lower predicted survival rates (**Figure 4** and **Table 2**).

**Figure 5:**
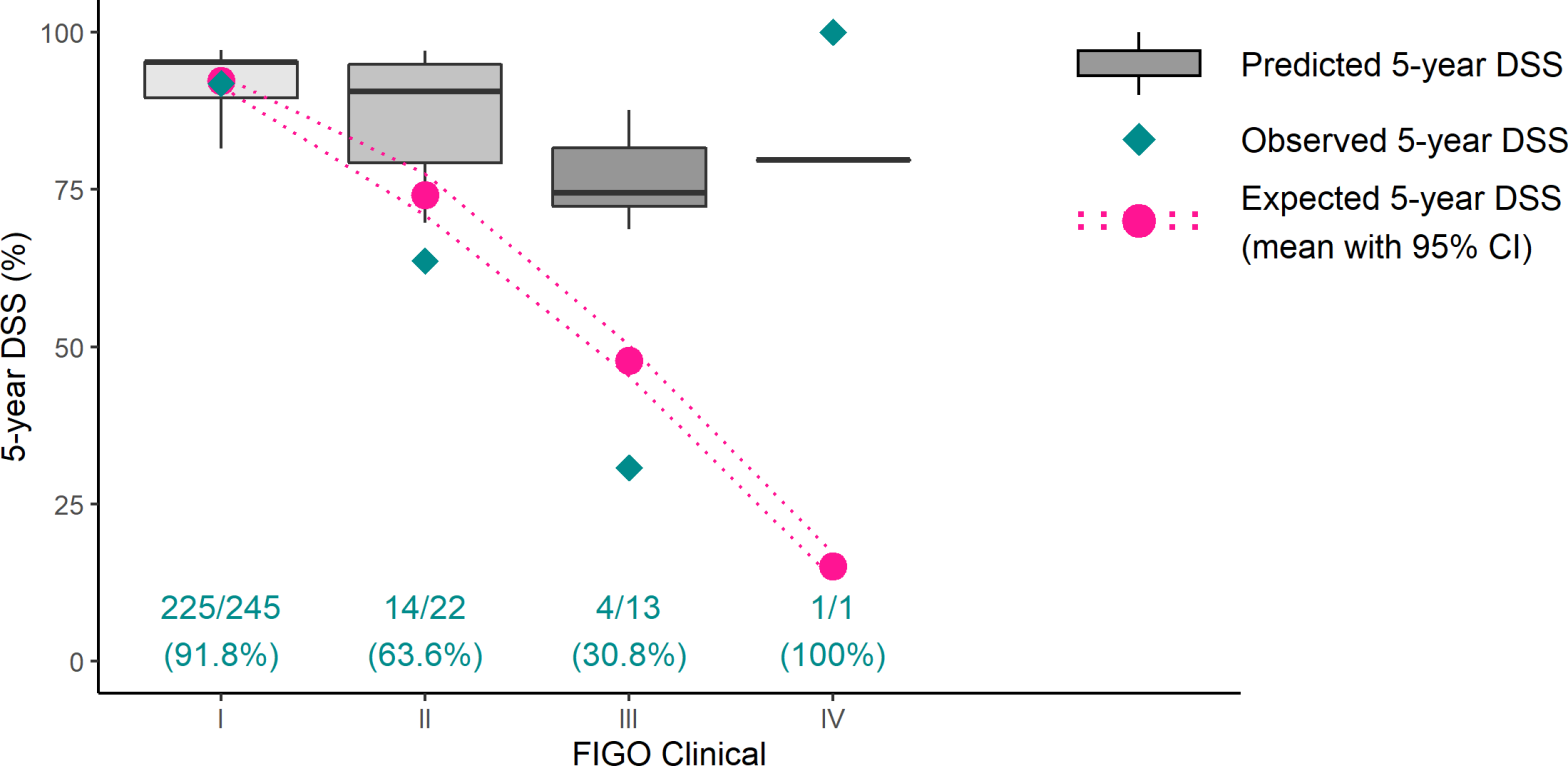
Five-year disease-specific survival prediction versus reality versus expectation in different clinical FIGO stages. Gray boxes represent the model’s prediction, green rhombuses indicate the real 5-year DSS, and pink dots denote expected 5-year survival according to surgical FIGO stages (10). Ideally, all green rhombuses lie in gray boxes. In clinical FIGO II–III stages, ENDORISK predicts much better survival than reality. Only one patient was preoperatively categorized into FIGO IV stadium—the survival result implies her misclassification. DSS, disease-specific survival; FIGO, International Federation of Gynecology and Obstetrics.

**Figure 5** displays the 5-year DSS prediction compared with reality and expected survival according to previously published probability (10) in different clinical FIGO stages (**Supplementary Figure 3** expands on surgical stages).

## Discussion

In the era of personalized medicine, we aim to have optimal information concerning a prognosis to facilitate adequate shared decision-making with the patient and define the most appropriate treatment decision. The ENDORISK model definitively contributes to the preoperative knowledge on risk of LNM and DSS.

Several EC predictive models have been published and focus on discriminating patients pre- and postoperatively into risk groups with predicting LNM or outcome. Previous models used traditional clinicopathological characteristics including LVSI, myometrial invasion, histotype, grade, age, and/or BMI (11, 12). So far, results were only moderate and, currently, additional immunohistochemical markers are already frequently used in the clinic: ER, PR, L1CAM, p53, Ki67 (13, 14). Some authors also included imaging information such as tumor diameter, myometrial/cervical invasion, or lymphadenopathy (15, 16).

The original multivariate analysis is based on a simple graphic calculating tool called nomogram. Jiang et al. published an LNM prediction model based on histological and IHC markers with a sensitivity of 82.8% and specificity of 82.7% (AUC 0.9) (14). However, this model cannot be applied when certain data are missing. Moreover, information on LVSI is required, which limits the use of the model in a preoperative setting. Similar results were presented with an effort to predict 3-year recurrence-free survival (sensitivity 76.5%, specificity 86.7%, AUC 0.82) with comparable limitations (including LVSI, all data required) (13).

With the development of computer technology, a Bayesian network has become more accessible, used for determining probable relationships and causalities based on expert knowledge with machine learning. An enormous advantage is that it can be applied, even when some patient characteristics are absent, which often occurs in clinical practice. The ENDORISK model was established with a variety of pre- and postoperative information, yet it could be applied exclusively with preoperative data. Minimally required data to work properly include (1) preoperative tumor grade, (2) minimally three of four IHC markers (ER, PR, p53, or L1CAM), and (3) at least one clinical biomarker (CA 125 serum level, LN status according to imaging method, platelet count, or pap smear result) (7).

The original model was created cognizant of histologic results from pelvic and para-aortic LN staging. Nowadays, complete lymphadenectomy is not the standard practice with all patients, and less invasive SNB is recommended with a low/intermediate-risk disease (3). Certain authors prefer this method even in high-risk cases (17). Isolated para-aortic nodal metastasis (notwithstanding negative pelvic nodes) occurs in approximately 1% of surgically staged cases (18). Consequently, we decided to also include patients with only pelvic dissection or SNB, reflecting current diagnostic practice. Sensitivity analysis, excluding SNB cases, presented comparable results, supporting the results of the complete study cohort (**Supplementary Figure 1**).

Historically, knowing the potential preoperative risk of LNM guided whether or not para-aortic-pelvic lymphadenectomy was indicated. Currently, SNB is preferred not only in low- but also in high-risk EC and might reduce the benefit of preoperative risk stratification. Yet, based on the very low risk in EC patients without myometrial invasion, LN staging could be omitted in these cases (3). If patients with truly low risk of LNM (<5%) could be properly identified preoperatively by using the ENDORISK model, SNB could be safely omitted in those hospitals where this technique is not available. An interesting question is whether it is necessary to provide LN staging in all EC types or if, according to other preoperative markers, we could abandon it. In an era of SNB staging practice, the ENDORISK model for LNM prediction could be used in hospitals, where this method is not available. Additionally, it could be supportive if SNB fails and side-specific lymphadenectomy is considered, especially in obese and fragile patients.

Our LNM prediction results were comparable with validation on MoMaTEC cohort: AUC 0.84 versus 0.82, Brier score 0.11 versus 0.09. The model very precisely predicts LNM in early stages, albeit underestimates clinically advanced carcinomas (**Figure 3**). For example, in patients with preoperative suspicion of LNM according to imaging methods, the ENDORISK model estimated an average probability of only 51% (25–78%). In fact, all were finally LNM positive. This might be explained by the low number of advanced cases; however, the model should be able to predict even worse stages.

ENDORISK model validation for 5-year DSS with our cohort displayed very similar results with previous cohorts MoMaTEC and PIPENDO, evaluated as well adjusted according to AUC (0.82, 0.84) and Brier score (0.12, 0.10) (7). Nevertheless, when using calibration plots to visualize the results, predictive value was obviously outstanding only for low-risk patients and significantly overrated for high-risk patients. This phenomenon was even more evident when patients were classified according to clinical FIGO stages (**Figure 5**). Definitely, the most accurate results were achieved, when the final surgical stage was applied (**Supplementary Figure 3**); nevertheless, this information is unknown preoperatively.

The FIGO stage is an important independent factor affecting survival, even during molecular classification times. The average 5-year survival is declining from 92% in stage I, 74% within stage II, and 48% in stage III to only 15% in stage IV (10). The ENDORISK model, currently, does not include information about the clinical stage disease (except for “enlarged lymph nodes on imaging”), even though, there are other possibilities for attaining these data. An expert oncogynecologic ultrasound or magnetic resonance imaging (MRI) is suitable for myometrial and cervical invasion detection; a CT scan can identify distant metastasis (19). Although myometrial invasion <50% of >50% is incorporated in the ENDORISK network, it is currently based on final histology, yet might be a very valuable addition to the model when determined preoperatively by either ultrasound or MRI. In addition, ultrasound-measured tumor-free distance from the tumor to the uterine serosa is another promising marker for predicting deep myometrial invasion and poor prognosis (20), which might be incorporated in an updated version of the network.

Even when we situate the worst clinical and histological characteristics into the model, the lowest survival prediction was 66%. This seems not in line with the published survival data of only 48%/15% in stage III/IV (10). Nevertheless, the number of cases with advanced stage in our cohort was limited and, hence, validation in larger cohorts is needed.

ENDORISK is one of the most complex risk stratification models so far. The authors imperiously searched the literature for potential relevant risk factors and assigned them statistically significant prognostic values. Unlike other models, ENDORISK could be applied even with strictly preoperative and incomplete information. However, as we ascertained, there is a need for further improvement before introduction into clinical practice. Clinical FIGO stage extension would definitively increase the model’s accuracy. Additionally, the incorporation of molecular classification would be highly relevant and is currently prepared in the ENDORISK 2.0.

Forthwith, we present the first unicentric ENDORISK model validation study, indicating a capacity for consistent treatment decisions and high-quality follow-up data. Innovatively, we have confirmed its application with SNB cases. Furthermore, we have suggested certain ancillary improvements to achieve better results among advanced cases that need to be considered when updating the ENDORISK network.

## Conclusions

ENDORISK is one of the best and most complex preoperative risk stratification models promulgated at this point in time. Nevertheless, there is still a place for improvement, particularly with survival prediction. Including clinical FIGO staging would increase model accuracy in advanced disease cases. In this SNB era, preoperative LNM predictive importance is waning; however, since SNB is not yet standard procedure in all countries, ENDORISK could be a helpful factor in decision-making regarding lymphadenectomy. With molecular classification’s inclusion into clinical practice, the ENDORISK model’s authors should consider its incorporation as well.

## Data availability statement

The original contributions presented in the study are included in the article/**Supplementary Material**. Further inquiries can be directed to the corresponding author.

## Ethics statement

The studies involving human participants were reviewed and approved by The Ethics Committee, University Hospital Brno, Brno, Czech Republic. The patients/participants provided their written informed consent to participate in this study.

## Author contributions

Conceptualization, PV, PO, and VW; study design, PV, PO, VW, and JP; statistical analysis, PO and PL; pathological examination, JH; writing – original draft preparation, PV, VW, and PO; writing – review and editing, JP, JH, PL, SV, and CR; supervision, VW and JP. All authors have read and agreed to the published version of the manuscript.

## Funding

This research was supported by the Ministry of Health of the Czech Republic, grant number NU21-09-00031 and the Development of Research Organization (FNBr, 65269705).

## Acknowledgments

We would like to thank Rich Zimmerman for manuscript proofreading and editing.

## Conflict of interest

The authors declare that the research was conducted in the absence of any commercial or financial relationships that could be construed as a potential conflict of interest.

## Publisher’s note

All claims expressed in this article are solely those of the authors and do not necessarily represent those of their affiliated organizations, or those of the publisher, the editors and the reviewers. Any product that may be evaluated in this article, or claim that may be made by its manufacturer, is not guaranteed or endorsed by the publisher.
