# Supplementary material for: External validation study of endometrial cancer preoperative risk stratification model (ENDORISK)
Source: Front Oncol. 2022 Aug 3;12:939226. doi: 10.3389/fonc.2022.939226 (PMC9381832; doi:10.3389/fonc.2022.939226)
Supplement: Supplementary file 1 [file DataSheet_1.docx]

Supplementary Material

# Supplementary Tables

|  | LNM (sensitivity analysis) |
| --- | --- |
| AUC (95% CI) | 0.82 (0.74-0.89) |
| Brier score | 0.14 |
| Predicted no. of events | 28.2 |
| Observed no. of events | 36 |
| Predicted/observed ratio (95% CI) | 0.78 (0.49-1.07) |

**Supplementary Table 1**: Sensitivity Analysis Concordance Statistics.

Excluding patients with sentinel node biopsy as a staging method provided very similar results to the main analysis. The discriminative performance was quantified based on AUC (a higher AUC implies better performance). Overall performance of the model was quantified by the Brier score (a lower Brier score signifies better accuracy of probabilistic predictions.

The predicted/observed ratio <1 denotes a lower prediction than reality, whereas a ratio >1 indicates overestimation compared to reality. If 95% CI includes value 1, the difference is non-significant.

AUC = area under the curve, CI = confidence interval, DSS = disease specific survival, LNM = lymph node metastasis

# Supplementary Figures


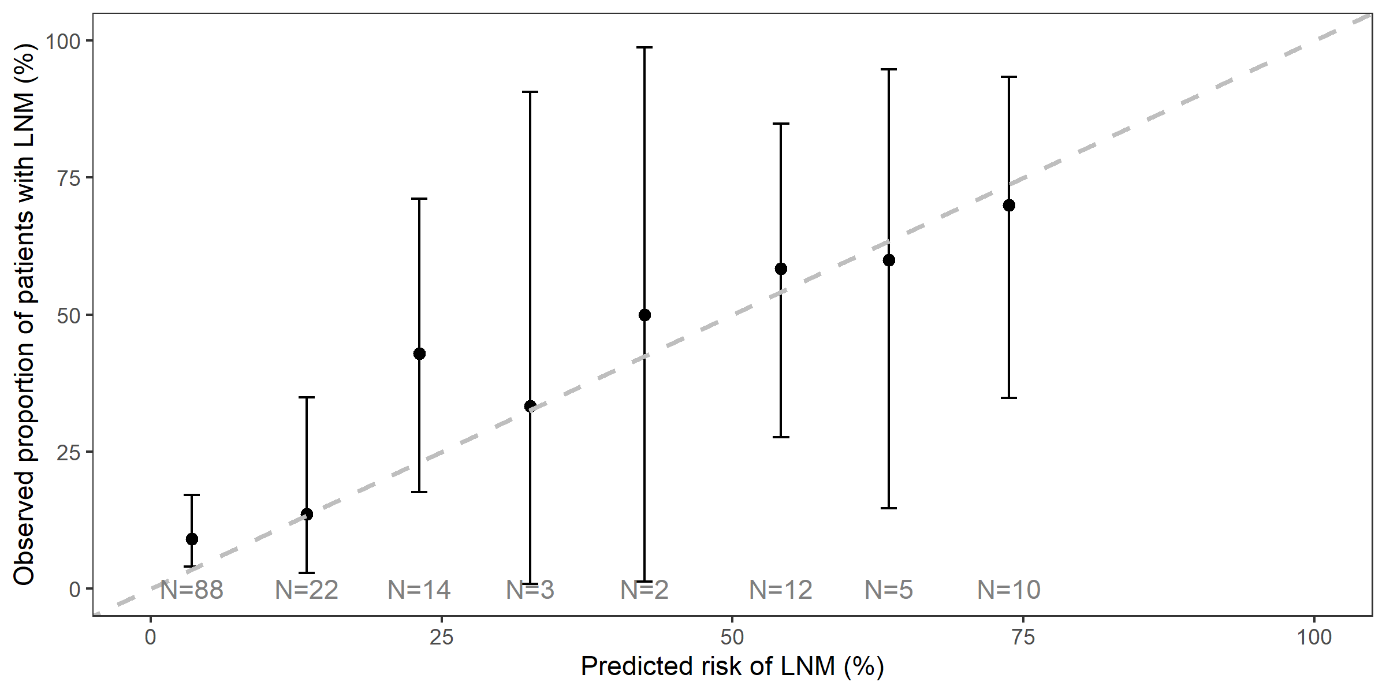


**Supplementary Figure 1**: Sensitivity Analysis LNM Calibration Plot of Observed vs Predicted Events

A dashed line displays the predicted value, and black dots represent the observed LNM. Ideally, all black marks are lying on the dashed line. Excluding SNB cases from the LNM cohort gives very similar results as the main analysis indicating proper inclusion without worsening predictive success.

LNM = lymph node metastasis


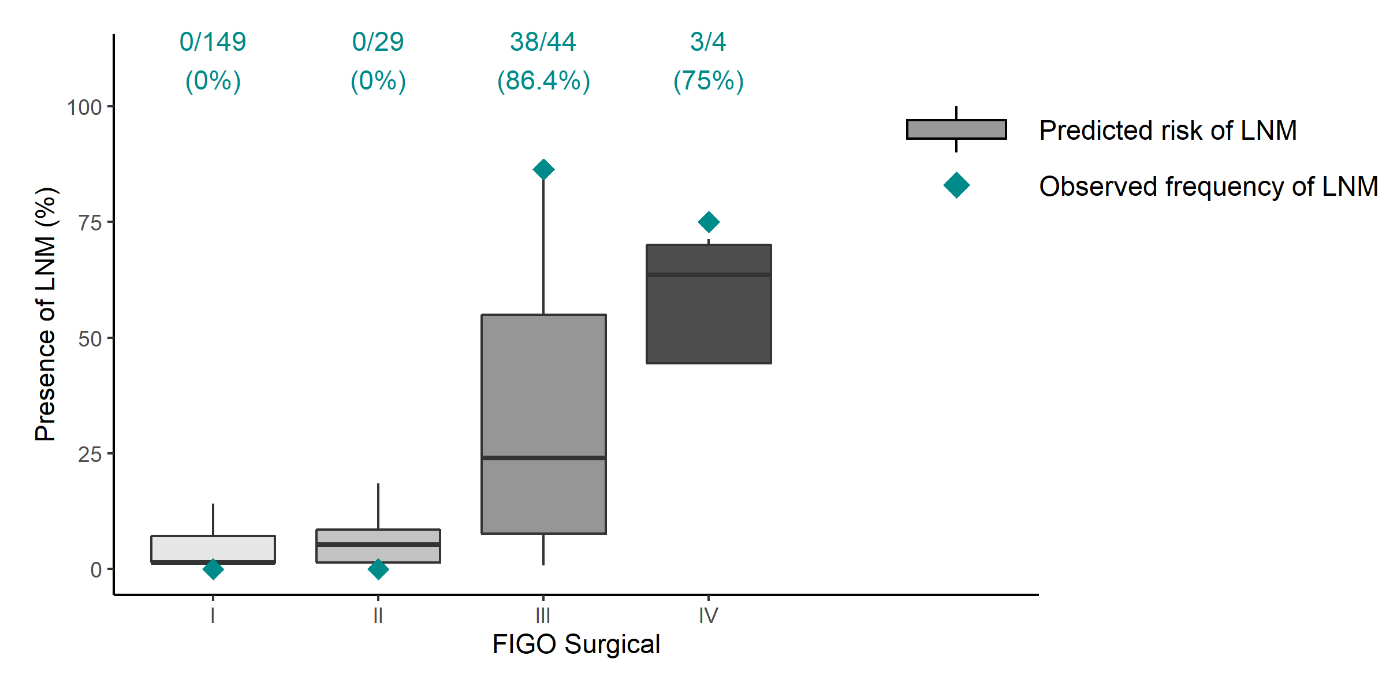


**Supplementary Figure 2**: Lymph Node Metastasis Prediction vs Reality in Different Surgical FIGO Stages

Grey boxes represent the model's prediction, green rhombuses indicate the real LNM frequency. Ideally, all green rhombuses lie in grey boxes. In the surgical FIGO III stage, ENDORISK predicts fewer cases of LNM than reality.

LNM = lymph node metastasis, FIGO = International Federation of Gynecology and Obstetrics


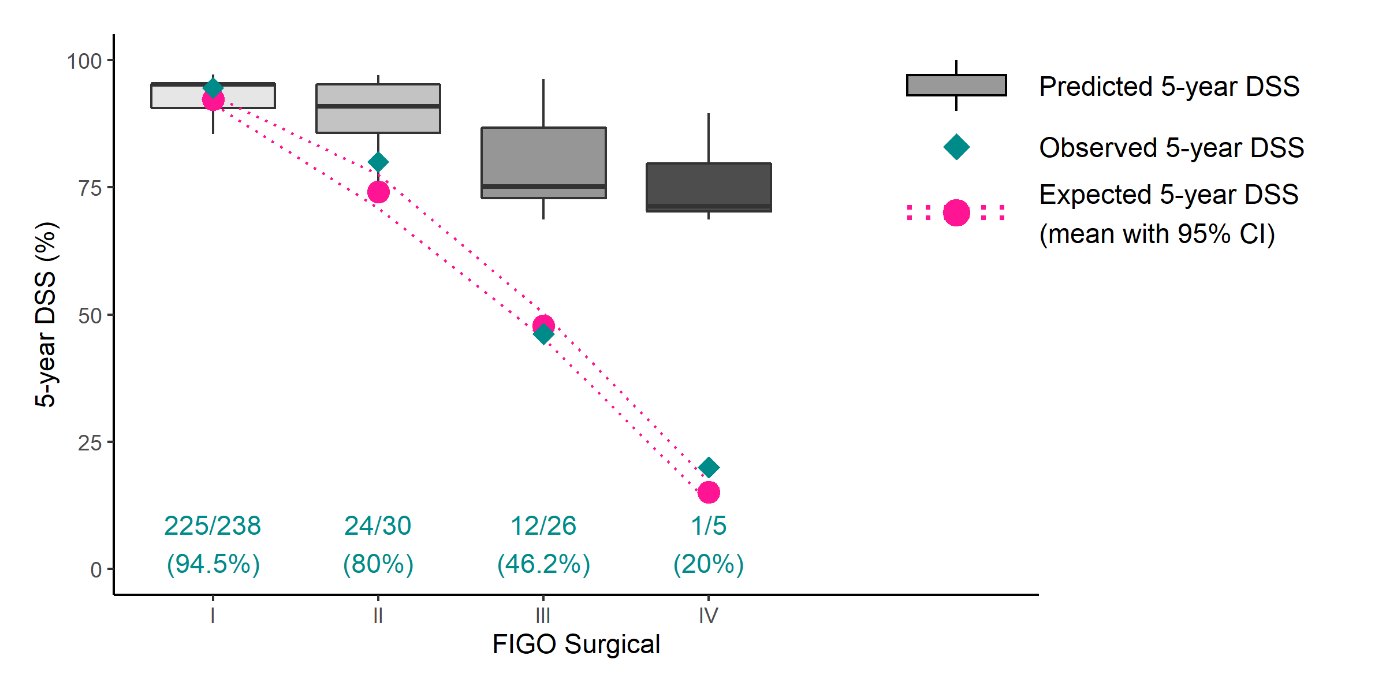


**Supplementary Figure 3:** 5-year Disease-specific Survival Prediction vs Reality vs Expectation in Different Surgical FIGO Stages

Grey boxes represent the model's prediction, green rhombuses denote the real 5-year DSS in our cohort, and pink dots suggest expected 5-year survival according to surgical FIGO stages^11^. Ideally, all green rhombuses lie in grey boxes. Our results were almost identical to expected survival (according to previously published survival), meanwhile the ENDORISK model in surgical FIGO III-IV stages predicts much better survival than reality.

DSS = disease-specific survival, FIGO = International Federation of Gynecology and Obstetrics
